# The geographical patterns of distribution of the genus *Teuthraustes* Simon, 1878 in Ecuador and description of three new species (Scorpiones, Chactidae)

**DOI:** 10.3897/zookeys.721.21529

**Published:** 2017-12-12

**Authors:** Eric Ythier, Wilson R. Lourenço

**Affiliations:** 1 SynTech Research, 613 route du Bois de Loyse, 71570 La Chapelle de Guinchay, France; 2 Muséum national d’Histoire naturelle, Sorbonne Universités, Institut de Systématique, Evolution, Biodiversité (ISYEB), UMR7205-CNRS, MNHN, UPMC, EPHE, CP 53, 57 rue Cuvier, 75005 Paris, France

**Keywords:** Amazon, biodiversity, Ecuador, geographical distribution, new species, Pacific, scorpion, taxonomy, *Teuthraustes*

## Abstract

Three new species of scorpions belonging to the genus *Teuthraustes* Simon, 1878 (Scorpiones: Chactidae) are described from the Amazonian and Pacific regions of Ecuador. The new descriptions raise to four the number of *Teuthraustes* species in Ecuadorian Amazonia and raise to two the number of species described from the Pacific region. The total number of species of *Teuthraustes* is now 27, including 15 in Ecuador. The geographical distribution of the genus in Ecuador is enlarged and its pattern of distribution in the country is also commented upon.

## Introduction

The genus *Teuthraustes* was created by Simon (1878) based on a new species, *Teuthraustes atramentarius* Simon, 1878 collected in Ecuador by M. Deville of the Brussels Museum. Both the genus and species descriptions are extremely reduced and poorly diagnosed. Between Simon’s (1878) description and the revision of the genus by Kraepelin (1911), thirteen additional species were described or transferred to *Teuthraustes*. With the exceptions of *T. amazonicus* (Simon, 1880) and *T. glaber* Kraepelin, 1912, both described from Peru, all other species are known from Ecuador. Even if the taxonomic status of some Ecuadorian species is still uncertain, this remarkable concentration of species in Ecuador is realistic and can be explained by biogeographic models (Lourenço 1995a).

From the revision by Kraepelin (1911) until the monograph work of Mello-Leitão (1945) no new species of this genus were described. Subsequently, five new species were described from Venezuela, *T. carmelinae* Scorza, 1954, *T. adrianae* González-Sponga, 1975, *T. akananensis* González-Sponga, 1984, *T. maturaca* González-Sponga, 1991 and *T. reticulatus* González-Sponga, 1991, three from Brazil, *T. lisei* Lourenço, 1994, *T. braziliensis* Lourenço & Duhem, 2010 and *T. newaribe* Lourenço, Giupponi & Pedroso, 2011, one from Colombia, *T. guerdouxi* Lourenço, 1995 and one from Peru, *T. castiglii* Rossi, 2015 (Lourenço 1994a, 1995b, Lourenço and Duhem 2010, Lourenço et al. 2011, González-Sponga 1996, Rossi 2015). Almost all the species of *Teuthraustes*, so far described, were collected in the Andean mountains in Ecuador and Colombia, and in the Amazonian highlands of Venezuela and Brazil. These highlands are known as the ‘Tepuys’. Exceptions remain however: *T. amazonicus* Simon, described from the region of Pebas, located on the banks of the Solimões River in Peruvian Amazonia, then later found in Brazilian and Colombian Amazonia, *T. glaber* Kraepelin and *T. castiglii* Rossi, both also described from the region of Pebas, Peru (to notice that the precise locality of *T. glaber* was originally indicated as only Peru, however in the personal notes by Simon the type specimen is indicated as from the region of Pebas), *T. braziliensis* Lourenço & Duhem, described from Brazilian Amazonia, *T. dubius* Borelli and *T. festae* Borelli, described from Morona-Santiago Province in Ecuador, *T. rosenbergi* Pocock, described from Guayas Province in Ecuador, and finally the three new species described in the present paper. In a recent publication by Brito and Borges (2015), an attempted checklist of the scorpions from Ecuador was proposed. This contribution is a compilation of previous works and no new species or locations were added neither to this fauna in general nor to the genus *Teuthraustes* in particular.

In the present paper, three new species of *Teuthraustes* are described from the lowlands of Ecuador: two from the Amazon region (Orellana and Sucumbíos Provinces; raising to four the number of *Teuthraustes* species in Ecuadorian Amazonia) and one from the rainforests of the Pacific Coast in Esmeraldas Province, this last species being the second record of a *Teuthraustes* species from the Pacific coastal region. The patterns of distribution presented by the species of the genus *Teuthraustes* in Ecuador are also discussed. This group remains, however, typical of highland formations of South America.

## Geographical pattern of distribution of the genus *Teuthraustes*

The known geographical distribution of the genus *Teuthraustes* clearly indicates its endemic and disrupted nature. Of the 27 species known at present, nine are distributed in the Andean highlands of Ecuador. Another group of seven species has been described even more recently from a different highland site located between Brazil and Venezuela. This area clearly corresponds with the Imeri endemic centre which has been defined both for plants and for animals (Lourenço 2001, Prance 1982a, b). It is located in the ‘Tepuys’ region which lies mainly in Guayana, a floristic province that has been delineated botanically (Mori 1991). One species is known from the highlands of Colombia, 8 species are known from the lowlands of the Ecuadorian, Peruvian, Colombian and Brazilian Amazonia and 2 species are known from the Pacific coast region of Ecuador.

This outstanding concentration of *Teuthraustes* species in the Andean highlands and in the Imeri endemic centre may be similar to the ‘explosive’ pattern of speciation proposed by Gentry (1992) for plants of the genus *Gasteranthus*. According to Gentry (1992), it is probable that an entirely different evolutionary mode may be operating in these areas. The exceedingly dynamic speciation is perhaps mediated more by genetic transilience associated with genetic drift in small founder populations in a kaleidoscopically changing milieu, than by fine-tuned selection of the type generally suggested to be typical of lowland rain forest.

Several botanists (Gentry 1988, 1992) and entomologists (Erwin 1982, Wilson 1988) agree that tropical rainforests are the most species-rich ecosystems on earth. They also agree, on the basis of solid evidence, that the ‘epicentre’ of global biodiversity occurs in the tropical Andes, a region of the upper Amazonia which includes the North of Peru, Ecuador and the southern half of Colombia. This suggestion seems to be valid for plants, vertebrates and butterflies (Prance 1982a, b, Gentry 1992, Erwin 1982, Wilson 1988). The region is also one of the world’s greatest sites of alpha-diversity on scorpions (Lourenço 1994b). Consequently, the very high concentration of *Teuthraustes* species in the Andean region could perhaps be no more than the consequence of the great ecological diversity there.

**Figure 1.**
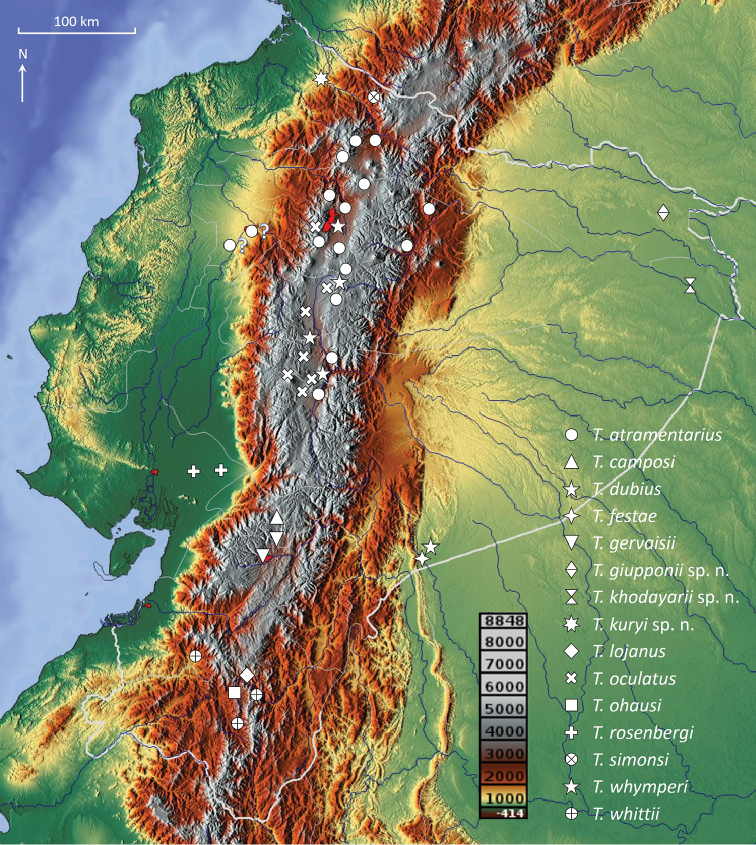
Topographic map of Ecuador showing the distribution of the known species of *Teuthraustes*.

It is obvious that scorpion speciation and differentiation is by no means recent. As stated by Haffer (1982), the isolation of large populations due to Tertiary palaeogeographic changes undoubtedly played a major role in establishing the basic distributional and evolutionary patterns of the tropical floras and faunas at higher taxonomic levels of family and genera. At the same time, members of less ‘plastic’ groups with low evolutionary rates in the present flora and fauna survived relatively unchanged since Tertiary times. Since the Andes arose in the form of strings of growing islands from a marine geosynclinal basin, there was no pre-Andean continuous and widespread lowland fauna occupying what was later to become the Andes and their forelands. Consequently, Andean elements must have come from abroad. The ‘Tepuys’ region which includes the Imeri endemic centre, is located in the Precambrian Guiana Craton (or shield). From a geological point of view the ‘Tepuys’ are composed of sheer blocks of Precambrian sandstone and quartzite rocks. These ‘mesas’ are the remains of a huge sandstone plateau that once covered the granite basement complex between what is today the northern border of the Amazon Basin and the Orinoco, between the Atlantic coast and the Rio Negro (Lourenço and Duhem 2009).

Ecological, paleoclimatic, and palynologic data (Prance 1982b) indicate that the apparent ‘stability’ of present-day rainforests was interrupted by periods of climatic change through several dry/wet/dry episodes of the late Cenozoic period, and especially during more recent Pleistocene and Holocene epochs. During the earlier Quaternary period, temperate regions were glaciated. Cooler and drier conditions prevailed in the present tropical zones and reduced the rainforest to savannas or dry-forests except in localized regions where conditions of temperature and humidity allowed them to persist. During these glacial phases, more mesic species of scorpions, such as those of the genus *Teuthraustes*, and also of other mesic genera such as *Chactas* Gervais, 1844 and *Vachoniochactas* González-Sponga, 1978 probably experienced a more enlarged range of distribution than that observed today. With the return of the present interglacial phase these genera are again restricted to the highlands where mesic conditions prevail. Some species of the genera *Teuthraustes* and *Chactas*, however, probably evolved in and became adapted to the tropical forest when this one expanded and coalesced throughout the entire Neotropical lowlands (Lourenço 1995a, Lourenço et al. 2005). Consequently, one can expect new species of both genera still to be found in the lowlands of Amazonia.

**Figure 2.**
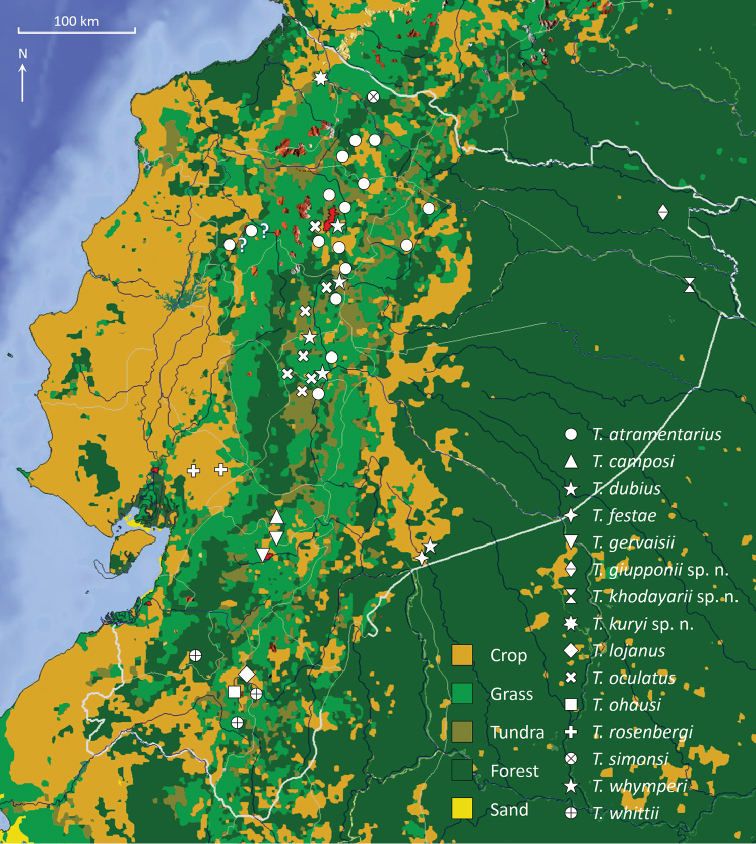
Vegetation map of Ecuador showing the distribution of the known species of *Teuthraustes*.

## Methods

Measurements and illustrations were made using a Wild M5 stereo-microscope with a drawing tube (*camera lucida*) and an ocular micrometre. Measurements follow those of Stahnke (1970) and are given in mm. Trichobothrial notations are those developed by Vachon (1974) and the morphological terminology mostly follows that of Hjelle (1990).

## Taxonomic treatment

### Family Chactidae Pocock, 1893

#### Genus *Teuthraustes* Simon, 1878

##### Composition of the genus *Teuthraustes*

*Teuthraustes adrianae* González-Sponga, 1975 – Venezuela (Amazonas)

*Teuthraustes akananensis* González-Sponga, 1984 – Venezuela (Amazonas)

*Teuthraustes amazonicus* (Simon, 1880) – Peru (Loreto), Brazil (Amazonas), Colombia (Amazonas)

*Teuthraustes atramentarius* Simon, 1878 – Ecuador (Chimborazo, Cotopaxi, Imbabura, Pichincha, Tungurahua)

*Teuthraustes braziliensis* Lourenço & Duhem, 2010 – Brazil (Amazonas)

*Teuthraustes camposi* (Mello-Leitão, 1939) – Ecuador (Cañar)

*Teuthraustes carmelinae* Scorza, 1954 – Venezuela (Amazonas)

*Teuthraustes castiglii* Rossi, 2015 – Peru (Loreto)

*Teuthraustes dubius* (Borelli, 1899) – Ecuador (Morona-Santiago)

*Teuthraustes festae* (Borelli, 1899) – Ecuador (Morona-Santiago)

*Teuthraustes gervaisii* (Pocock, 1893) – Ecuador (Azuay)

*Teuthraustes giupponii* sp. n. – Ecuador (Sucumbíos)

*Teuthraustes glaber* Kraepelin, 1912 – Peru (Loreto)

*Teuthraustes guerdouxi* Lourenço, 1995 – Colombia (Boyaca)

*Teuthraustes khodayarii* sp. n. – Ecuador (Orellana)

*Teuthraustes kuryi* sp. n. – Ecuador (Esmeraldas)

*Teuthraustes lisei* Lourenço, 1994 – Brazil (Amazonas)

*Teuthraustes lojanus* (Pocock, 1900) – Ecuador (Loja)

*Teuthraustes maturaca* González-Sponga, 1991 – Venezuela (Amazonas)

*Teuthraustes newaribe* Lourenço, Giupponi & Pedroso, 2011 – Brazil (Amazonas)

*Teuthraustes oculatus* Pocock, 1900 – Ecuador (Chimborazo, Cotopaxi, Tungurahua)

*Teuthraustes ohausi* Kraepelin, 1912 – Ecuador (Loja)

*Teuthraustes reticulatus* González-Sponga, 1991 – Venezuela (Amazonas)

*Teuthraustes rosenbergi* (Pocock, 1898) – Ecuador (Guayas)

*Teuthraustes simonsi* (Pocock, 1900) – Ecuador (Carchi)

*Teuthraustes whymperi* (Pocock, 1893) – Ecuador (Cotopaxi, Pichincha, Tungurahua)

*Teuthraustes wittii* (Kraepelin, 1896) – Ecuador (Loja)

###### Teuthraustes khodayarii sp. n.

Taxon classificationAnimaliaScorpionesChactidae

http://zoobank.org/0F55C3BE-7744-4872-B55A-6B39F7960325

**Figure 3.**
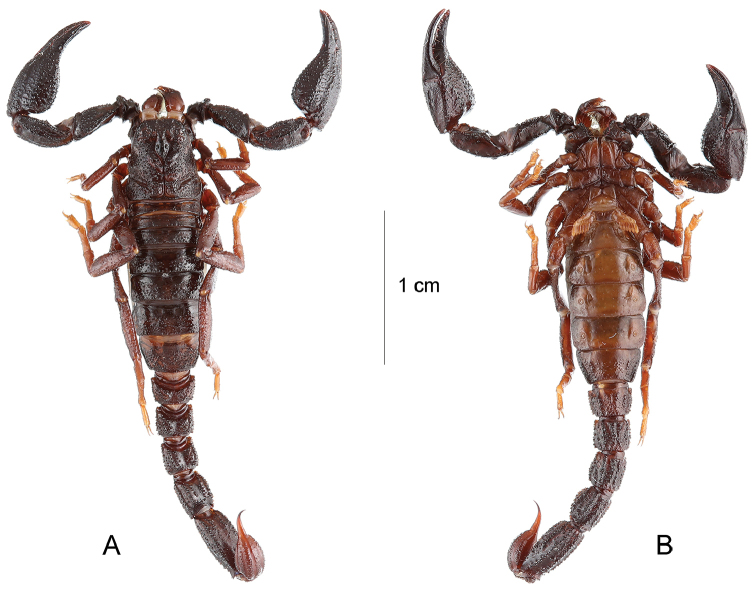
*Teuthraustes khodayarii* sp. n. female holotype.. Habitus, dorsal **A** and ventral **B** aspects.

Fig. 3 , 4 , Table 1

####### Type material.

Ecuador, Orellana Province (formerly Napo), Santa Maria de Huiririma 0°43'0"S, 75°37'0"W) VI/1976 (British expedition and local people), rainforest, under log. Pre-adult female holotype. Type material deposited in the Muséum national d’Histoire naturelle, Paris, France.

####### Etymology.

The specific name honours Dr. Khosro Khodayari, founder, President and CEO of SynTech Research, Davis, California, USA, in recognition of his support for the study of scorpions.

####### Diagnosis.

Moderate to small scorpion with 35.1 mm in total length. Colouration reddish brown to dark brown. Body and appendages very intensely granulated with a rather thin granulation. Pectines with 6–6 teeth in female. Fixed and movable fingers of chela with 5–5 rows of granules; extremities of fingers with 3 granules. Ventral carinae present on metasomal segments I to IV, but weak on I. Trichobothrial pattern of type C neobothriotaxic ‘majorante’.

####### Description.

Based on female holotype.

####### Colouration.

General colouration basically reddish brown to dark brown. Prosoma: carapace reddish brown with a few paler spots; eyes blackish. Mesosoma: tergites reddish brown, with VII slightly paler with some yellow spots; venter and sternites reddish yellow; pectines and genital operculum with the same colour as sternites. Metasoma: segments reddish brown, with darker zones over carinae; vesicle reddish yellow with the tip of the aculeus dark red. Chelicerae: reddish yellow, with some diffuse variegated brown spots; fingers dark red with teeth reddish yellow. Pedipalps: dark brown with blackish carinae; cutting edges of fingers reddish. Legs: reddish yellow to reddish brown without spots.

####### Morphology.

Prosoma: carapace intensely granulated, with a dense and thin granulation; furrows shallow; sternum pentagonal, wider than long. Mesosoma: tergites almost acarinate, with a thin and dense granulation; sternites smooth and shiny, only VII with a few granulations; spiracles oval to rounded-shaped and conspicuous; pectinal tooth count 6–6, fulcra vestigial. Metasoma: segments I to III wider than long; metasomal tegument on segments I to IV with conspicuous granulations; segment V with spinoid granulations ventrally; carinae on segments I–V strongly developed; ventral present on all segments, less marked on I; telson with strongly marked granulations, only dorsal face smooth; aculeus shorter than vesicle. Chelicerae: dentition typical of the family Chactidae (Vachon 1963), and with dense setation ventrally and internally. Pedipalps: femur with dorsal internal, dorsal external and ventral internal carinae strongly marked; ventral external carina absent; dorsal, ventral and internal aspects with granulations; patella granulated with moderate carinae; chela very strongly granulated; internal aspect with conspicuous granules; dentate margins on movable and fixed fingers with 5–5 rows of granules; distal edge of fingers with three granules; trichobothriotaxy of type C; neobothriotaxic ‘majorante’ (Vachon 1974). Legs: tarsi with short setae disposed in a single line.

####### Morphometric values.

Female holotype of *T. khodayarii* sp. n. Total length (in mm) including telson, 35.1. Carapace: length, 5.3; anterior width, 3.1; posterior width, 5.4. Mesosoma length 11.1. Metasomal segments. I: length, 1.9; width, 3.0; II: length, 2.0; width, 2.7; III: length, 2.2; width, 2.5; IV: length, 2.7; width, 2.3; V: length, 4.8; width, 2.2; depth, 2.0. Telson length, 5.1; vesicle: width, 2.0; depth, 1.6. Pedipalp: femur length, 3.8, width, 1.3; patella length, 3.7, width, 1.8; chela length, 8.1, width, 2.9, depth, 2.8; movable finger length, 4.6.

####### Relationships.

The species can be distinguished from the others congeners in particular from *Teuthraustes atramentarius* Simon, which is also distributed in Ecuador, but exclusively in the high central Andes, by the following features: (i) a thin but very intense granulation over body and appendages, particularly marked on legs (ii) ocular tubercle much less pronounced, (iii) pectines are wider, (iv) pedipalpal chela are not bulk and elongated, (v) metasomal segments I to III wider than long.

**Figure 4.**
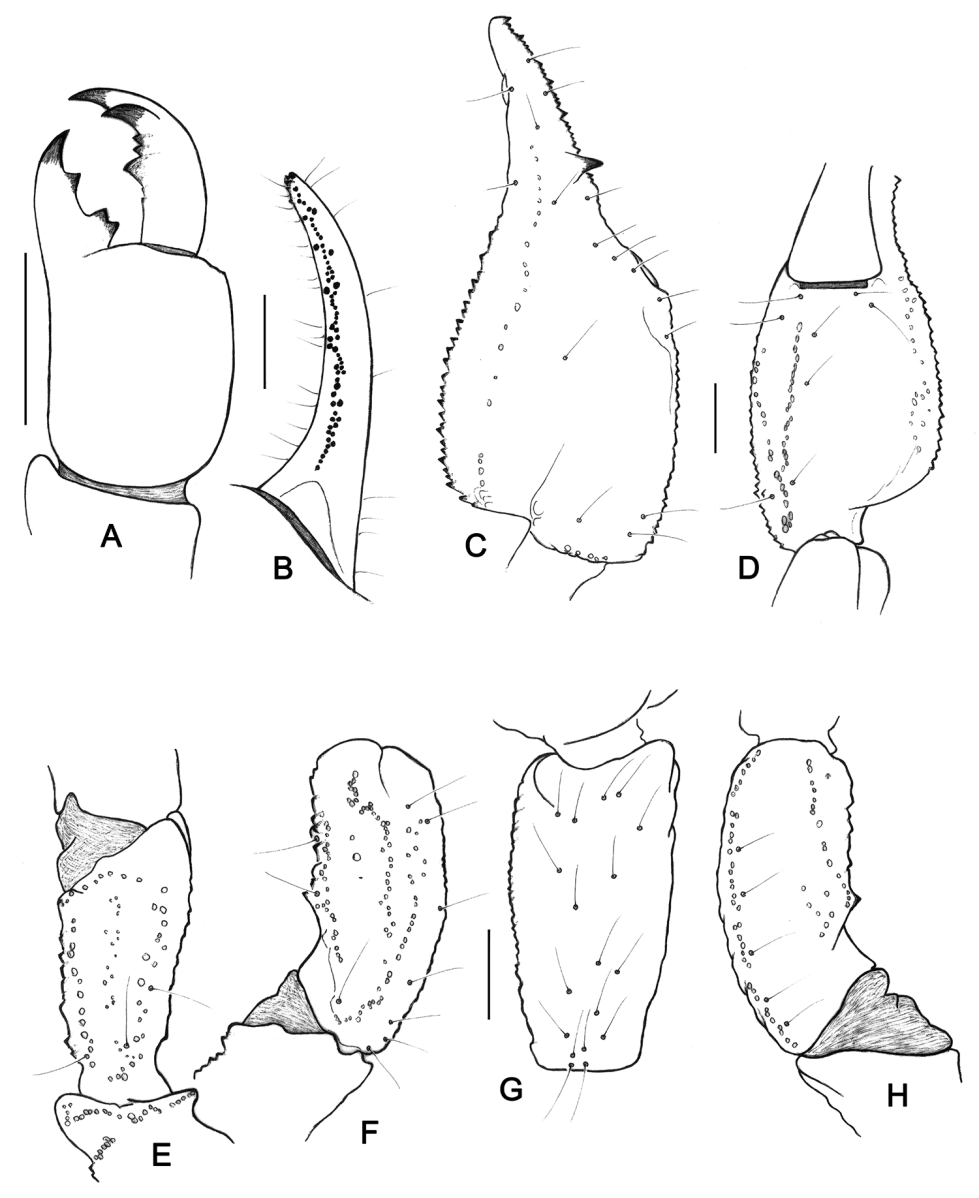
*Teuthraustes khodayarii* sp. n. Female holotype. **A** Chelicera, dorsal aspect **B** Disposition of the granulation over the dentate margins of pedipalp-chela movable finger **C–H** Trichobothrial pattern **C–D** Chela, dorso-external and ventral aspects **E** Femur, dorsal aspect **F–H** Patella, dorsal **F** external **G** and ventral **H** aspects. Scale bars 1 mm.v

**Table 1.** Morphometric values (in mm) of female holotypes of *T. khodayarii* sp. n., *T. giupponii* sp. n., *T. kuryi* sp. n., and *T. atramentarius* Simon, 1878.

	*T. khodayarii*	*T. giupponii*	*T. kuryi*	*T. atramentarius*
Total length	35.1	52.1	60.4	48.9
Carapace:				
length	5.3	7.1	8.0	7.1
anterior width	3.1	4.3	4.8	4.3
posterior width	5.4	8.0	8.6	7.3
Mesosoma length	11.1	15.5	16.2	14.8
Metasoma length	18.7	29.1	36.2	27.0
Metasomal segment I:				
length	1.9	3.0	3.6	2.7
width	3.0	4.0	4.9	3.8
Metasomal segment II:				
length	2.0	3.3	4.1	3.1
width	2.7	3.6	4.6	3.5
Metasomal segment III:				
length	2.2	3.6	4.6	3.3
width	2.5	3.6	4.5	3.5
Metasomal segment IV:				
length	2.7	4.3	5.6	4.0
width	2.3	3.4	4.3	3.3
Metasomal segment V:				
length	4.8	7.3	9.4	6.7
width	2.2	3.1	4.2	3.2
depth	2.0	2.7	3.6	2.8
Telson:				
length	5.1	7.6	8.9	7.2
width	2.0	3.5	4.3	3.3
depth	1.6	2.8	3.6	2.9
Pedipalp:				
femur length	3.8	4.7	5.7	4.8
femur width	1.3	2.1	2.4	1.9
patella length	3.7	5.6	6.1	5.3
patella width	1.8	2.5	2.8	2.9
chela length	8.1	11.2	11.1	10.7
chela width	2.9	4.0	5.1	3.7
chela depth	2.8	5.3	7.2	5.6
Movable finger length	4.6	6.3	5.9	5.8

###### Teuthraustes giupponii sp. n.

Taxon classificationAnimaliaScorpionesChactidae

http://zoobank.org/64DE0CC3-8C83-4B2A-820F-50B6B4D8102D

**Figure 5.**
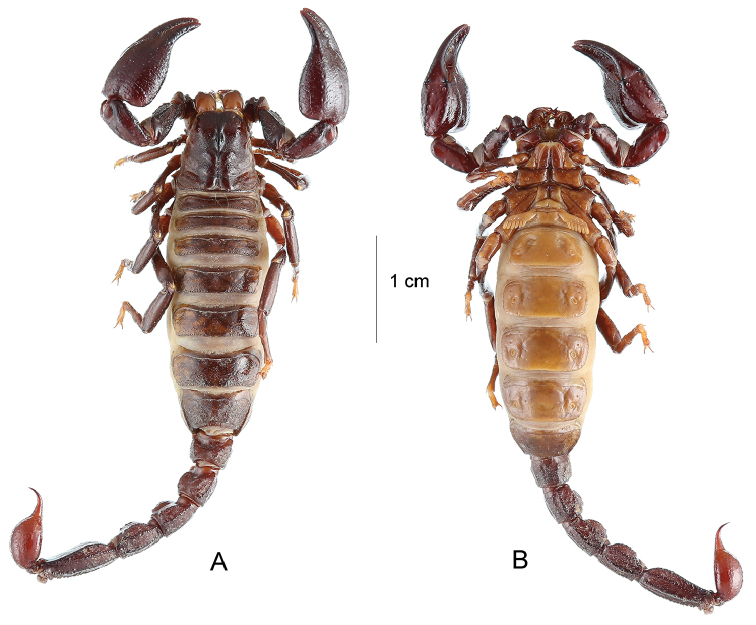
*Teuthraustes giupponii* sp. n. Female holotype. Habitus, dorsal **A** and ventral **B** aspects.

**Figure 6.**
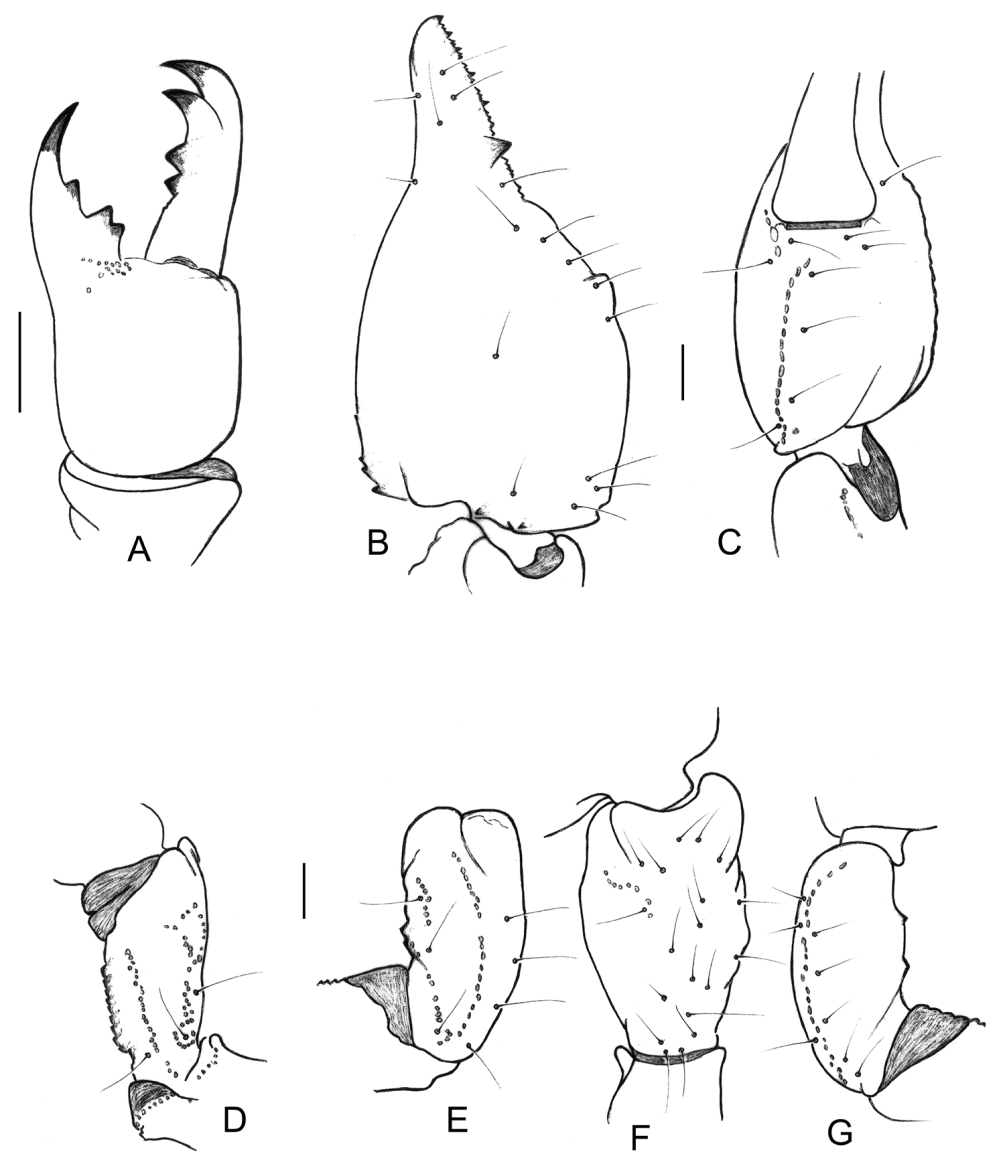
*Teuthraustes giupponii* sp. n. Female holotype. **A** Chelicera, dorsal aspect **B–G** Trichobothrial pattern **B–C** Chela, dorso-external and ventral aspects **D** Femur, dorsal aspect **E–G** Patella, dorsal **E** external **F** and ventral **G** aspects. Scale bars 1 mm.

Figs 5 , 6 , Table 1

####### Type material.

Ecuador, Sucumbíos Province (formerly Napo), Dureno/Cuyabeno, near lake Cuyabeno, Cuyabeno Wildlife Reserve (0°07'00"S, 75°50'00"W) VI/1976 (British expedition and local people), rain-forest, under stone. Female holotype. Type material deposited in the Muséum national d’Histoire naturelle, Paris, France.

####### Etymology.

The specific name honours Dr. Alessandro Ponce de Leão Giupponi, Fundação Oswaldo Cruz, Rio de Janeiro, for his contributions to the study of scorpions.

####### Diagnosis.

Moderate scorpions with 52.1 mm in total length. Colouration reddish to reddish yellow. Body and appendages weakly granulated, with minute punctation. Pectines small with 6–6 teeth in female. Fixed and movable fingers of chela with 5–5 rows of granules. Ventral carinae present on metasomal segments I to IV but inconspicuous on I. Trichobothrial pattern of type C neobothriotaxic ‘majorante’.

####### Description.

Based on female holotype.

####### Colouration.

General colouration basically reddish to reddish yellow. Prosoma: carapace reddish; eyes blackish. Mesosoma: tergites reddish yellow, paler than carapace, with confluent yellowish strips; venter and sternites reddish yellow to yellow; pectines, genital operculum, and sternites yellow. Metasoma: segments reddish, with darker zones over carinae; vesicle reddish yellow with the tip of the aculeus dark red. Chelicerae: yellow, with some diffuse variegated reddish spots at the base of fingers; fingers reddish yellow; teeth reddish. Pedipalps: dark reddish; carinae blackish. Legs: reddish yellow with pale spots.

####### Morphology.

Prosoma: carapace acarinate, with dense minute granulations; lateral edges smooth; furrows shallow; sternum pentagonal, wider than long. Mesosoma: tergites acarinate, with a few granulations and some punctations; sternites, smooth and shiny; spiracles oval to rounded-shaped and conspicuous; VII acarinate with a few granules; pectinal tooth count 6–6, fulcra inconspicuous. Metasoma: segments I and II wider than long; metasomal tegument on segments I to IV with strong granulations; segment V with some spinoid granulations ventrally; carinae on segments I–V strongly developed; ventral present on segments I to IV; telson with a few ventral granulations, other faces almost smooth; aculeus shorter than vesicle. Chelicerae: dentition typical of the family Chactidae (Vachon 1963), and with dense setation ventrally and internally. Pedipalps: femur with dorsal internal, dorsal external and ventral internal carinae strongly marked; ventral external carina represented by a few granules; dorsal, ventral and internal aspects with thin granulations; patella smooth with weak carinae; chela smooth and almost acarinate; internal aspect with a few small granules; dentate margins on movable and fixed fingers with 5–5 rows of granules; trichobothriotaxy of type C; neobothriotaxic ‘majorante’ (Vachon 1974). Legs: tarsi with short setae disposed in a single line.

####### Morphometric values.

Female holotype of *T. giupponii* sp. n. Total length (in mm) including the telson, 52.1. Carapace: length, 7.5; anterior width, 4.3; posterior width, 8.0. Mesosoma length 15.5. Metasomal segments. I: length, 3.0; width, 4.0; II: length, 3.3; width, 3.6; III: length, 3.6; width, 3.6; IV: length, 4.3; width, 3.4; V: length, 7.3; width, 3.1; depth, 2.7. Telson length, 7.6; vesicle: width, 3.5; depth, 2.8. Pedipalp: femur length, 4.7, width, 2.1; patella length, 5.6, width, 2.5; chela length, 11.2, width, 4.0, depth, 5.3; movable finger length, 6.3.

####### Relationships.

The new species can be distinguished from other the congeners in particular from *Teuthraustes oculatus* Pocock, which is also distributed in Ecuador, but in the high central Andes, by the following features: (i) paler general colouration, reddish to reddish yellow, (ii) tergites and pedipalps weakly granulate to smooth, (iii) carapace with some thin granulations (iv) lateral eyes small and apart from each other, (v) anterior edge of carapace not straight, lobate, (vi) ventral carinae on metasomal segment I inconspicuous.

###### Teuthraustes kuryi sp. n.

Taxon classificationAnimaliaScorpionesChactidae

http://zoobank.org/DB39E8D8-51EB-4784-AECF-43FC3BE4A923

**Figure 7.**
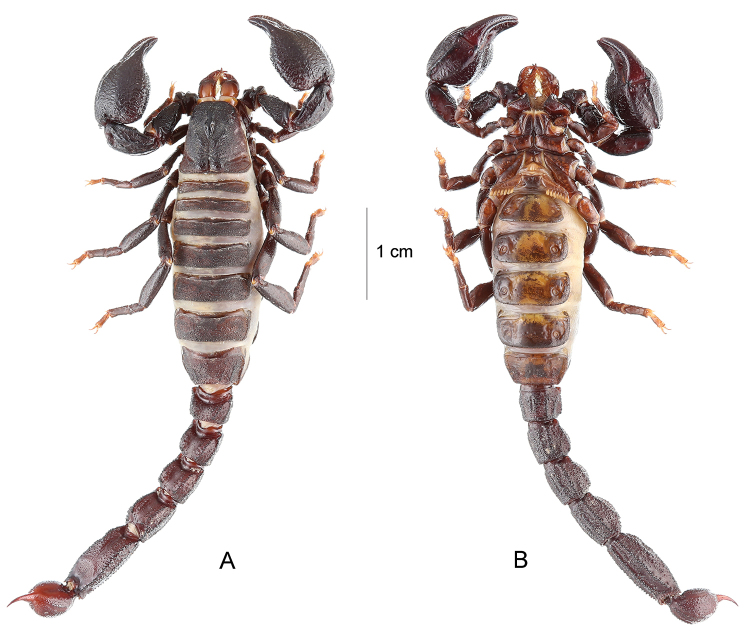
*Teuthraustes kuryi* sp. n. Female holotype. **A–B** Habitus, dorsal **A** and ventral **B** aspects.

Fig. 7 , 8 , Table 1

####### Type material.

Ecuador, Esmeraldas Province (N00.88883, W078.53732), 873 m, in zone where rainforest was recently destroyed, under log, 17/III/2011 (A. Chagas, A. Giupponi, A. Kury, M. Vega). Female holotype and female paratype. Holotype deposited in the Museo de Zoologia, Pontificia Universidad Católica de Quito, Ecuador; paratype deposited in the Museu Nacional, Rio de Janeiro, Brazil.

####### Etymology.

Specific name honours Prof. Adriano B. Kury, Museu Nacional, Universidade Federal do Rio de Janeiro, Brazil, for his important contribution to the study of arachnids.

####### Diagnosis.

Moderate to large scorpions with 60–61 mm in total length. Colouration dark brown to blackish. Body and appendages strongly granulated. Pectines with 7–7 teeth in females. Pedipalps bulk with short fingers; fixed and movable fingers of chela with 5–6 rows of granules. Metasoma long and strong; ventral carinae strongly marked on metasomal segments I to IV. Trichobothrial pattern of type C neobothriotaxic ‘majorante’.

####### Description.

Based on female holotype and female paratype.

####### Colouration.

General colouration basically dark brown to blackish. Prosoma: carapace dark brown; eyes blackish. Mesosoma: tergites reddish brown to dark brown, slightly paler than carapace; venter and sternites reddish brown to dark brown; pectines and genital operculum yellow. Metasoma: segments blackish brown, with darker zones over carinae; vesicle reddish brown with the base of the aculeus reddish yellow. Chelicerae: reddish yellow, with some diffuse variegated reddish spots at the base of the fingers; fingers dark reddish. Pedipalps dark brown to blackish; carinae blackish. Legs dark brown to blackish.

####### Morphology.

Prosoma: carapace almost acarinate, but with dense strongly marked granulations on the entire surface, except on the zone of furrows; furrows deep; sternum pentagonal, wider than long. Mesosoma: tergites intensely granulated but less marked than carapace; sternites, smooth and shiny; spiracles oval-shaped and conspicuous; VII acarinate with granulations; pectinal tooth count 7–7 in both females, holotype and paratype, fulcra vestigial. Metasoma: segments I and II wider than long; metasomal tegument on segments I to IV strongly granulated including dorsal aspect; segment V with some spinoid granulations ventrally; carinae on segments I–V strongly developed; ventral present on all segments; telson strongly granulated; aculeus shorter than vesicle. Chelicerae: dentition typical of the family Chactidae (Vachon 1963), and with dense setation ventrally and internally. Pedipalps: femur with dorsal internal, dorsal external and ventral internal carinae strongly marked; ventral external carina vestigial; dorsal, ventral and internal aspects granulated; patella granulated with well-marked carinae; chela strongly granulated and with most carinae well-marked; dentate margins on movable and fixed fingers with 5–6 rows of granules; trichobothriotaxy of type C; neobothriotaxic ‘majorante’ (Vachon 1974). Legs: tarsi with short setae disposed in a single line.

####### Morphometric values.

Female holotype of *T. kuryi* sp. n. Total length (in mm) including the telson, 60.4. Carapace: length, 8.0; anterior width, 4.8; posterior width, 8.6. Mesosoma length, 16.2. Metasomal segments. I: length, 3.6; width, 4.9; II: length, 4.1; width, 4.6; III: length, 4.6; width, 4.5; IV: length, 5.6; width, 4.3; V: length, 9.4; width, 4.2; depth, 3.6. Telson length, 8.9; vesicle: width, 4.3; depth, 3.6. Pedipalp: femur length, 5.7, width, 2.4; patella length, 6.1, width, 2.8; chela length, 11.1, width, 5.1, depth, 7.2; movable finger length, 5.9.

####### Relationships.

The new species can be distinguished from the others congeners in particular from *Teuthraustes atramentarius* Simon, which is also distributed in Ecuador, but exclusively in the high central Andes, by the following features: (i) carapace, tergites, pedipalps, metasoma, and telson strongly granulated, (ii) metasomal segments I to V long and strong with well-marked ventral carinae, (iii) pedipalps bulk with short fingers on chela, (iv) ventral aspect with a darker pigmentation, reddish brown to dark brown.

**Figure 8.**
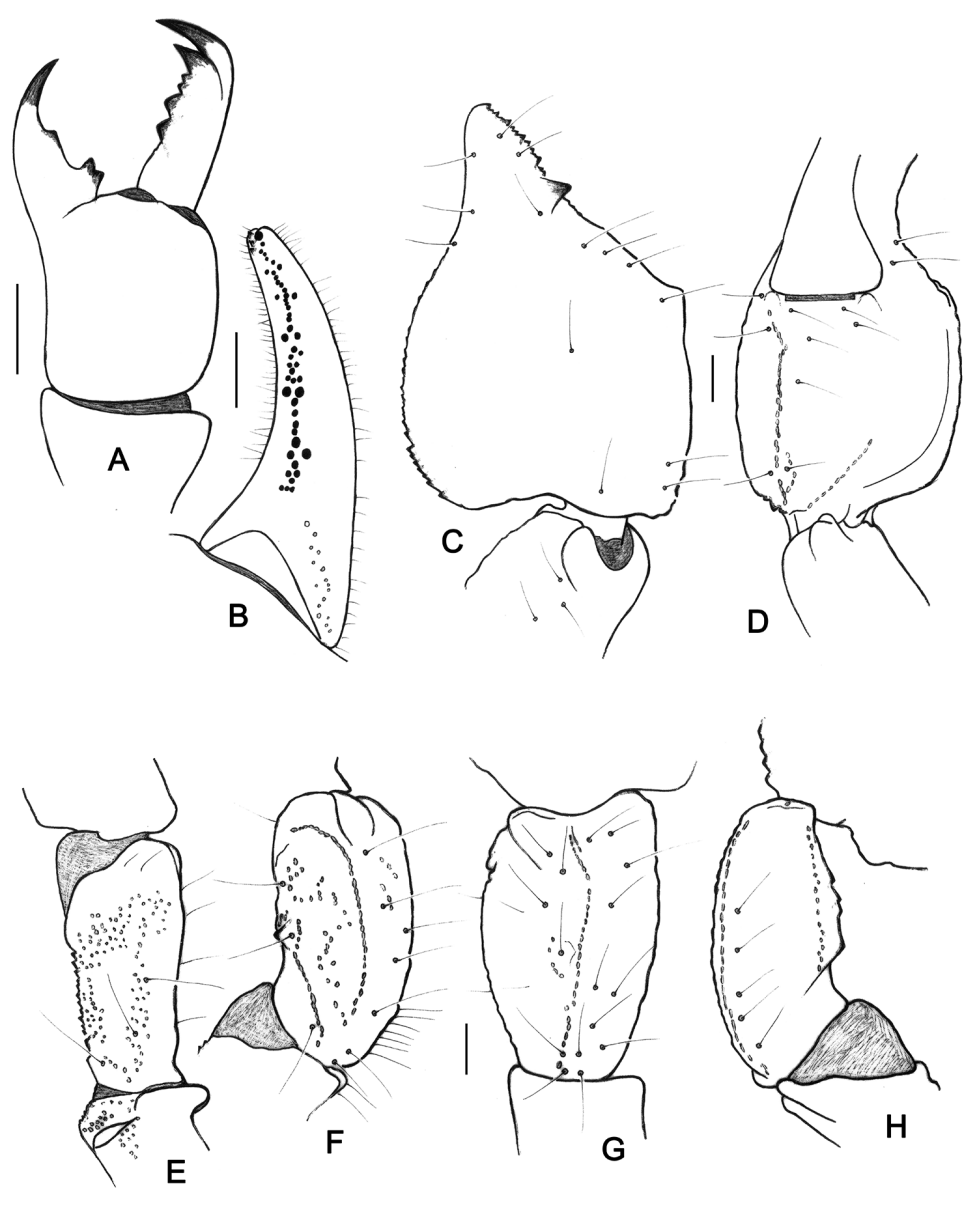
*Teuthraustes kuryi* sp. n. Female holotype. **A** Chelicera, dorsal aspect **B** Disposition of the granulation over the dentate margins of pedipalp-chela movable finger **C–H** Trichobothrial pattern **C–D** Chela, dorso-external and ventral aspects **E** Femur, dorsal aspect **F–H** Patella, dorsal **F** external **G** and ventral **H** aspects. Scale bars 1 mm.

###### *Teuthraustes atramentarius* Simon, 1878

The female holotype of *Teuthraustes atramentarius* was studied and its morphometric values (in mm) are presented in Table 1. The holotype is deposited in the Muséum national d’Histoire naturelle, Paris under the N° RS-0763 (Simon’s N° 3019). In the catalogue of the scorpions of the World (Sissom 2000) the holotype is cited as deposited in the “Musée Royal d’Histoire naturelle de Belgique” in Brussels. This is, however, incorrect.

**Figure 9.**
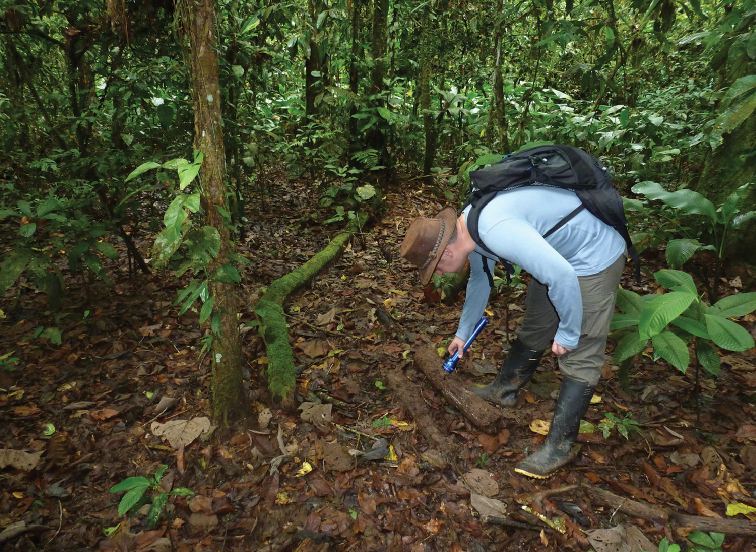
Amazon region, Ecuador. Natural habitat of *Teuthraustes khodayarii* sp. n. and *Teuthraustes giupponii* sp. n. (photograph C. Benros-Ythier; E. Ythier in the photo).

**Figure 10.**
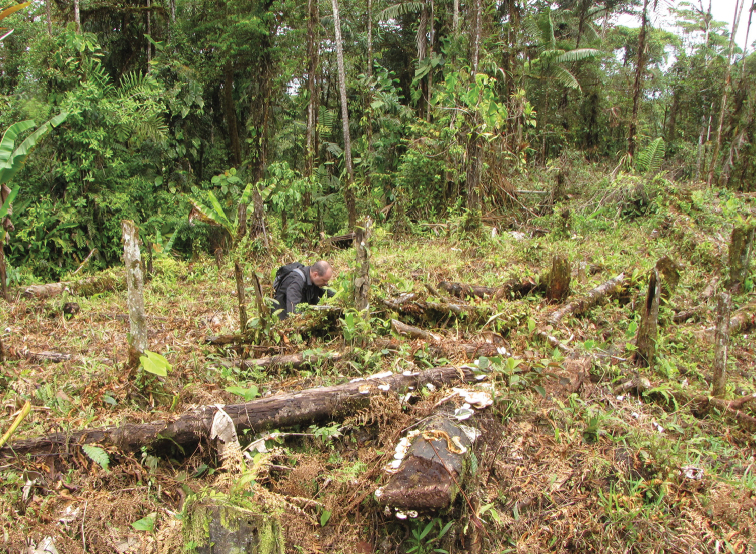
Esmeraldas Province, Ecuador. Natural habitat of *Teuthraustes kuryi* sp. n. (photograph A. Chagas Júnior; A.B. Kury in the photo).

## Supplementary Material

XML Treatment for Teuthraustes khodayarii

XML Treatment for Teuthraustes giupponii

XML Treatment for Teuthraustes kuryi
